# A Case Report and Literature Review: When Should We Treat the Seminal Vesicles in Prostate Cancer?

**DOI:** 10.7759/cureus.95670

**Published:** 2025-10-29

**Authors:** Rohail Syed, Nitin Vaishampayan, Ramesh Boggula, Michael Joiner, Dongping Shi, Steven R Miller

**Affiliations:** 1 Department of Oncology, Wayne State University School of Medicine, Detroit, USA

**Keywords:** conformal radiation therapy, ga-68 psma (gallium-68 prostate-specific membrane antigen), pelvic mri, prostate cancer, radiation therapy, seminal vesicle invasion

## Abstract

The most common cancer in men is prostate cancer. Treatment options for prostate cancer consist of radiation therapy, active surveillance, chemotherapy, and surgical intervention. In this case, radiation therapy was used, but it can lead to adverse effects ranging from fatigue, impacted sexual function, genitourinary side effects such as dysuria or nocturia, and gastrointestinal side effects such as nausea, abdominal pain, or diarrhea. Minimizing the treatment only to areas with PET-positive disease and those with a significant risk of microscopic involvement, therefore reducing radiation exposure, can help mitigate side effects and help maintain the patient’s quality of life. Here, we examine a man with prostatic adenocarcinoma. A prostate-specific membrane antigen (PSMA)-PET scan was used to determine if the seminal vesicles demonstrated any evidence of disease and if treatment of the area was necessary. A PSMA-PET scan showed invasion of the seminal vesicles; thus, the seminal vesicles were included as a target for radiation therapy. The patient tolerated the treatment with minimal side effects. This case reviews the indications for treating the whole seminal vesicle.

## Introduction

Approximately 12.5% of men receive a diagnosis of prostate cancer in their lifetime [1], and many undergo treatment with external beam radiation therapy (EBRT). Limiting the side effects of EBRT and maintaining an acceptable quality of life during and after treatment is essential. One way to help reduce side effects, especially rectal and urinary toxicity, is to minimize the volume of tissue exposed to EBRT and only treat the areas of disease, thereby potentially mitigating radiation toxicity. An article from the Prostate Cancer Research Institute notes that targeting the pelvic lymph nodes or seminal vesicles during radiation treatment increases the risk of short and long-term side effects to the small intestine, rectum, and bladder [2]. Prostate-specific membrane antigen (PSMA)-PET scan is a relatively new imaging technique, and studies have demonstrated that it may offer greater sensitivity and specificity in identifying pelvic nodal, seminal vesicle, and metastatic disease compared to conventional imaging [3]. Traditionally, the entire seminal vesicles are included in the radiation volume for high-risk prostate cancer patients, and the proximal 1 cm of the seminal vesicles for favorable intermediate risk prostate cancer patients. However, with new imaging techniques such as PMSA and MRI, it may be possible to avoid treating the entire seminal vesicles if the seminal vesicles are not involved with disease. To evaluate the extent of disease within the seminal vesicles, a PSMA-PET scan was used in conjunction with an MRI. Our patient had both positive seminal vesicle involvement on PSMA-PET scan and MRI. The patient underwent EBRT to the pelvis, prostate, and entire seminal vesicles. This case report evaluates the need to treat the seminal vesicles in patients without involvement of the seminal vesicles after imaging on PMSA and MRI.

## Case presentation

We present a case of a man in his 60s, who was noted to have an elevated prostate-specific antigen (PSA) of 7 ng/ml, three years before establishing care with radiation oncology. A PSA at the time of diagnosis was 13 ng/ml. The patient underwent initial imaging studies, including a nuclear medicine whole-body bone scan, which revealed no scintigraphic evidence for metastatic disease to the bone.

**Figure 1:**
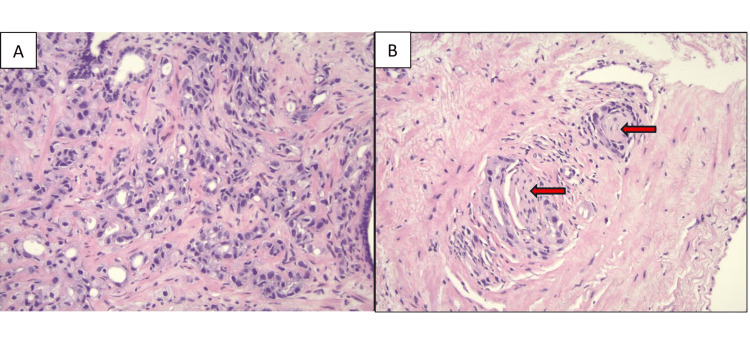
Image A (200x): The microphotograph reveals Gleason pattern 4 of prostatic adenocarcinoma. The tumor cells are arranged in ill-defined and fused glands. Image B (200x): The microphotograph displays tumor clusters within the perineural spaces of two nerves. The two nerves, indicated by the red arrows, are surrounded by tumor clusters.

A prostate biopsy demonstrated multifocal prostate adenocarcinoma, with the highest Gleason scores of 8 (4+4) in the right medial sector at the base of the prostate and the right lateral base. A total of 7/12 cores were positive, and a high percentage of the tumor volume in multiple biopsies consisted of a Gleason pattern 4, and perineural invasion was noted on the biopsy (Figure 1).

**Figure 2:**
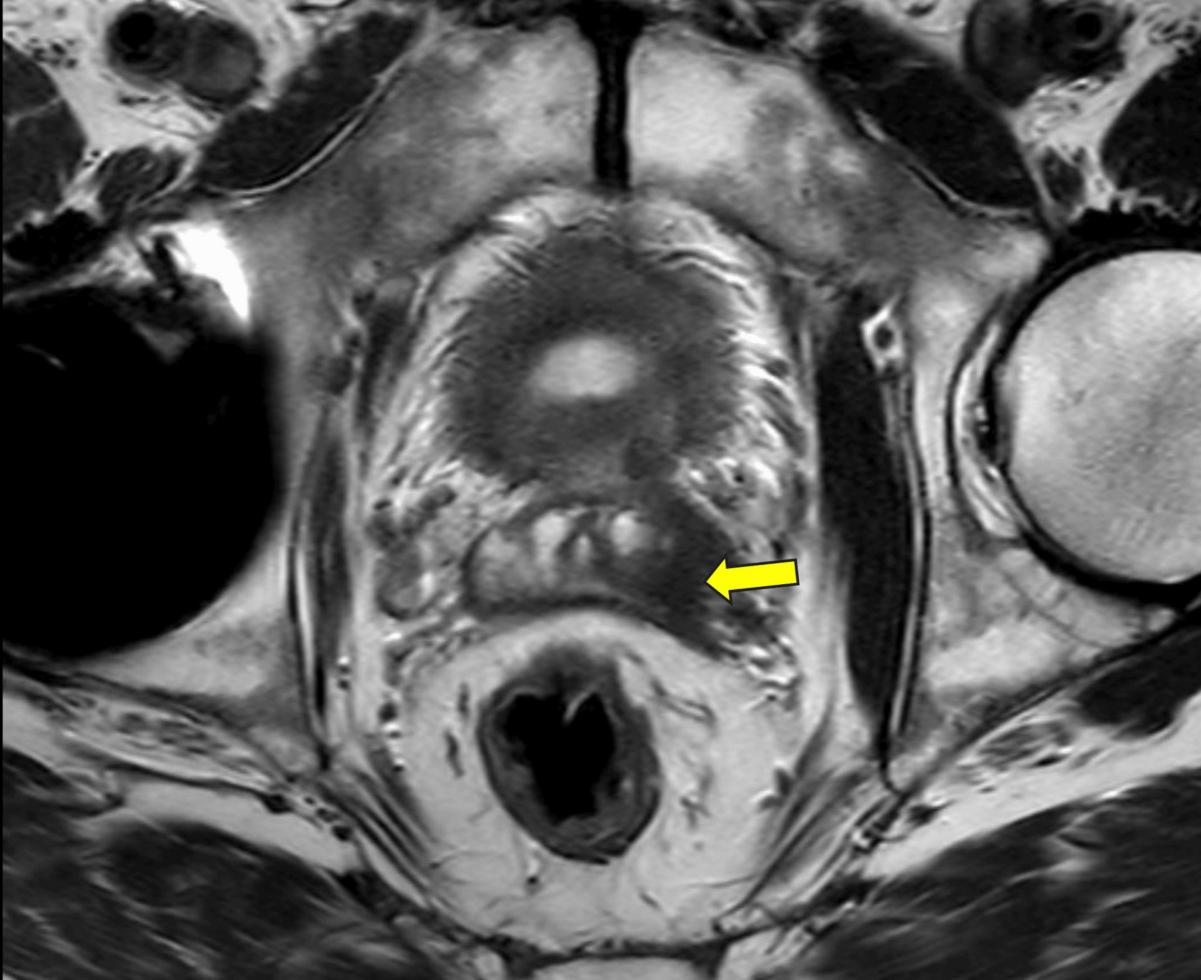
Axial T2 turbo spin echo (TSE) MRI image with the yellow arrow pointing to the hypo-intense area involving the left seminal vesicle, suspicious for disease.

Additional staging studies, including a T2 axial fast spin echo (FSE) prostate MRI scan with and without contrast, were performed, which revealed that the primary malignancy involved the majority of the left-sided peripheral gland, crossing the midline and extending into the right-sided peripheral zone at the 6-7 o'clock position, and affecting the central gland at the level of the mid-gland. There was a large area of capsular contact throughout the left-sided peripheral zone, with loss of a discrete capsular signal at the level of the mid-gland from the 2 to 5 o'clock position, which is highly concerning for extracapsular spread. The MRI also revealed malignant involvement of the proximal left seminal vesicle with no evidence of rectal or urinary bladder invasion. There were Prostate Imaging Reporting and Data System (PI-RADS) 2 changes involving the right-sided peripheral zone, which may represent benign etiology or low-grade prostate cancer, with a heterogeneous bone marrow signal with patchy enhancement. There was limited evaluation of the right-sided lower pelvis due to artifacts from the right hip arthroplasty (Figure 2).

**Figure 3:**
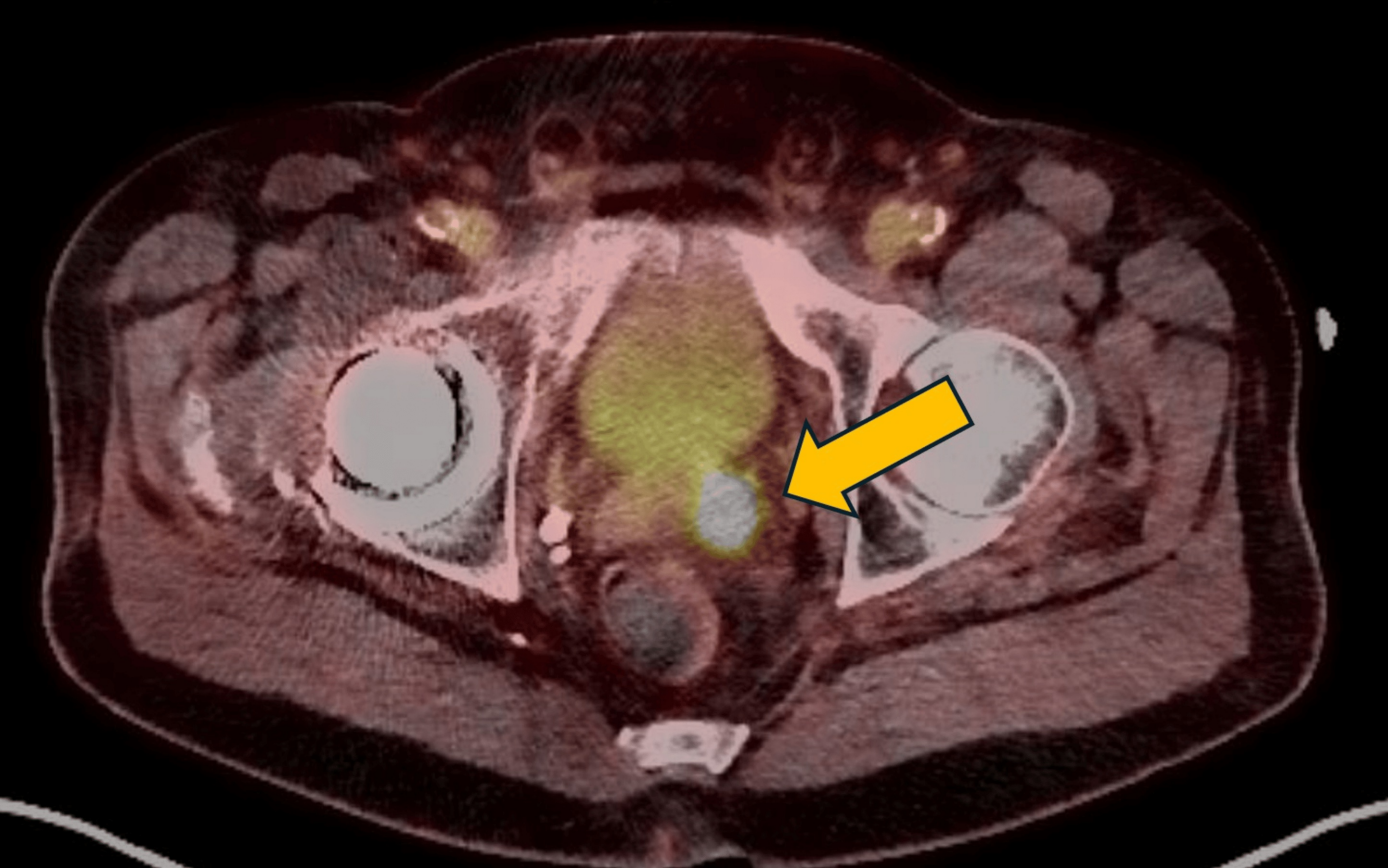
Axial prostate-specific membrane antigen (PMSA) scan displaying involvement of the left seminal vesicle.

A PSMA-PET/CT scan was performed, imaging the area from the skull to the thigh. The antigen Ga-68 gozetotide was administered via IV injection, and a standard uptake period was allowed before the CT and PET scans were performed. The imaging revealed an approximately 3.6 x 2.2 cm metabolically active mass involving the central and left peripheral zones, with direct tumoral extension into the left seminal vesicle (Figure 3).

**Figure 4:**
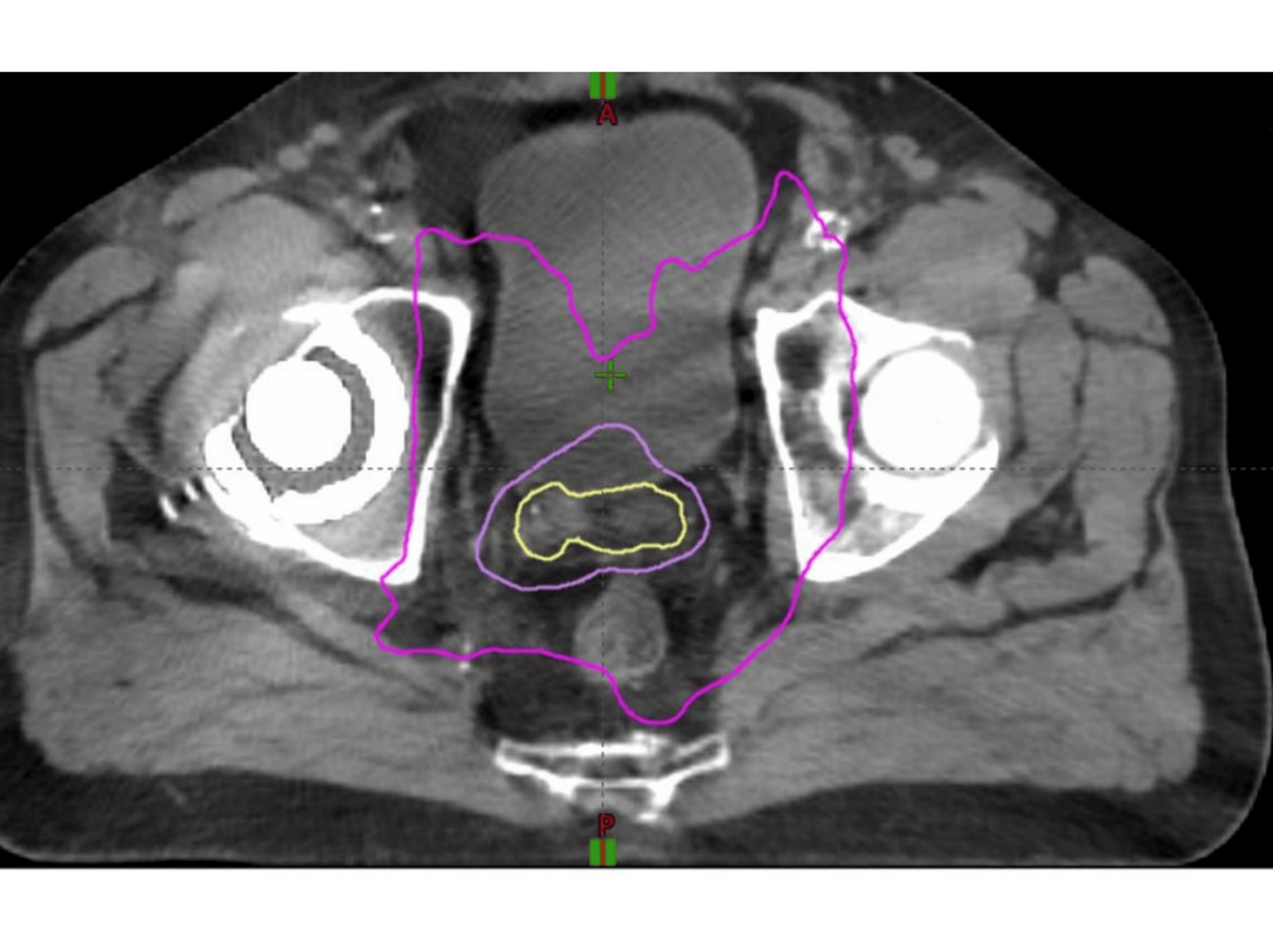
Axial image displaying the radiation treatment plan and isodose lines. Yellow is the 7420 cGy isodose line, violet is the 5040 cGy isodose line, and purple is the 6678 isodose line.

The patient underwent a course of EBRT and hormone therapy. The gross tumor volume (GTV) consisted of the lesion involving the prostate and the left seminal vesicle as noted on MRI and PMSA scans. No expansion was used for the planning target volume (PTV), which was treated to 7420 cGy. The clinical target volume (CTV) 5040 consisted of the internal, external, and common iliac lymph nodes, as well as the obturator lymph nodes. The PTV5040 cGy was a 5-7 mm expansion from the CTV. The CTV7000 consisted of the prostate and the full seminal vesicles. The PTV5040 was a 5 mm expansion around this to account for setup error and patient motion. He received a total dose of 5040 centigray (cGy) to the pelvic lymph nodes in 28 fractions and 7000 cGy to the prostate and entire seminal vesicle in 28 fractions using intensity modulated radiation therapy (IMRT). The PSMA-positive lesion in the prostate and the left seminal vesicle were treated to a total dose of 7420 cGy in 28 fractions (Figure 4).

During radiation therapy, he also underwent hormone therapy and received a Lupron injection of 22.5 mg. As of the patient’s three-month follow-up for his Lupron injection, he has not experienced any significant bowel or bladder issues. His most recent PSA following treatment was 0.33 ng/ml. There was no clinical or biochemical evidence of recurrent disease.

## Discussion

Per the European Society for Radiotherapy & Oncology (ESTRO) guidelines, it is recommended to treat the proximal 1.4 cm of the seminal vesicles in low risk (PSA ≤ 10 ng/ml, biopsy Gleason score ≤6 (Grade group 1), clinical stage ≤T2a and <50% of the biopsies involved) to intermediate risk (PSA > 10 and ≤20 ng/ml or Gleason score of 7 (Grade group 2 and 3) or clinical stage T2b) patients. However, treatment of the seminal vesicles in low-risk patients can be left to institutional policy. In high-risk patients (PSA > 20 ng/ml or Gleason score ≥8 (Grade group 4 and 5) or clinical stage ≥T2c), recommendations include treating the proximal 2.2 cm of the axial plane [4].

A study by Kestin et al. in 2002 examined the pathology of 344 radical prostatectomies, considered risk factors that could contribute to seminal vesicle invasion (SVI), and compared the results of the biopsy to these risk factors [5]. The high-risk factors considered in this study were pretreatment PSA, biopsy Gleason score, and clinical T classification. A low-risk patient was defined as having a PSA level less than 10 ng/mL, a Gleason score less than 6, and a clinical stage less than T2a. A value higher than the values listed was considered a high-risk factor, as it indicates a more aggressive disease. One percent of low-risk patients (31% of the population) showed seminal vesicle involvement. Patients with one high-risk feature (either having a PSA of 10 or greater, a Gleason score greater than or equal to 6, or a clinical stage of T2b or more) encompassed 38% of the population and had positive seminal vesicles at a rate of 15%. Patients with two high-risk features (20% of the population) had positive seminal vesicles 38% of the time, and patients with three high-risk features tested positive 58% of the time (p-values for all of these statistics were less than 0.001). Additionally, the median length of seminal vesicle involvement was 1 cm, with 90% of patients having 2 cm of involvement or less. The only two characteristics of the cancer that appeared to correlate with the length of seminal vesicle involvement were the pattern of spread and the percentage of gland involvement in the adenocarcinoma (p = 0.08). A discontinuous pattern of spread was associated with greater length in seminal vesicle involvement (p = 0.08). Aside from those two, no other characteristics seemed to be associated with seminal vesicle length. For this reason, the author recommends treating the proximal 2-2.5 cm of the seminal vesicle in patients with high-risk factors [5]. This study highlights significant factors to consider when determining the risk of a patient with prostate cancer and the chance of the disease spreading to the seminal vesicles. With respect to our patient, both his PSA and Gleason score would be considered high-risk factors, and according to Kestin et al., 2 cm of the proximal seminal vesicles should be treated. This study was done before PSMA imaging techniques were available, so we plan to examine the accuracy of PSMA imaging to determine if this imaging would allow PSMA to be used instead of treating the proximal 1-2 cm of the seminal vesicles.

Another study by Bayman et al. in 2007 reviewed multiple studies to determine independent factors that could contribute to SVI [6]. They examined four studies regarding prostate cancer and risk factors related to SVI, including Kestin et al., as well as a Mayo Clinic multivariate study covering over 3000 patients, and other retrospective analyses. From the paper, the factors most important in determining a patient's risk of SVI were pretreatment PSA, Gleason score, the clinical T stage, and the percentage of a positive core biopsy. The studies recommended excluding the seminal vesicles from the CTV "in patients with prostate cancer treated with definitive radiotherapy who have a pretreatment PSA≤10ng/ml, a biopsy Gleason score of 6 or less, stage≤T2a, and a percentage of positive biopsy≤50%." The risk of SVI in this group of patients is ≤ 5% [6]. This study provides another set of recommended guidelines, similar to those of Kestin et al., and it goes over multiple studies to corroborate their findings.

As mentioned previously, EBRT of the seminal vesicles can lead to adverse effects on the small intestine and otherwise expose patients to unnecessary radiation toxicity. A study done in 2023 by Steed et al. examined the cases of 359 patients with localized prostate cancer [7]. In accordance with ESTRO guidelines, they determined that no patient with low-risk prostate cancer should have any part of the seminal vesicle treated. The Memorial Sloan Kettering prostate cancer nomogram was used to assign a predicted risk of seminal vesicle involvement for each patient. To determine if seminal vesicle involvement was greater or less than 15%, they used the Memorial Sloan Kettering Cancer Center (MSKCC) pre-radical prostatectomy nomogram, which contains pretreatment data and long-term outcomes of over 10,000 prostate cancer patients, to perform dynamic statistical analysis using SPSS software. They considered the patient's age, PSA level, Gleason pattern, clinical tumor stage, and biopsy cores. They used these variables to determine the risk of lymph node involvement and the risk of seminal vesicle involvement based on the MSKCC database. They found that in those with a risk of seminal vesicle involvement greater than 15%, it was recommended that the seminal vesicles not be contoured in radiation therapy [7].

A literature review from Chow et al. in 2023 compared the diagnostic accuracy of PSMA-PET scans to conventional imaging techniques. The study examined 2431 patients who primarily had intermediate to high-risk prostate cancer (2.2% of the cohort contained patients with low-risk prostate cancer) across 31 different studies. The study found that PSMA-PET/MRI was 25.8% more sensitive than multiparametric MRI (mpMRI), which is a specialized MRI that employs techniques such as T2-weighted imaging and diffusion-weighted imaging to visualize the prostate better (p < 0.001) for extraprostatic extension (EPE) detection and 15.7% (p = 0.02) for SVI detection. However, there was no statistically significant difference found in the specificity of mpMRI and PSMA-PET/MRI imaging. Their study also found that PSMA-PET/CT appeared to be less than 9.6% sensitive than mpMRI (p = 0.2) for EPE detection and 16.9% less sensitive (p = 0.1) for SVI detection. When comparing PSMA-PET/MRI to mpMRI, the study examined 210 patients across four studies for EPE and 175 patients across three studies. When comparing PSMA-PET/CT to mpMRI, the study examined 228 patients across five studies for EPE and 518 patients across eight studies [8]. These results indicate that in patients with intermediate to high-risk prostate cancer, PSMA-PET used in conjunction with MRI is more sensitive than mpMRI when staging SVI and the spread of cancer beyond the prostate gland.

Additionally, regarding the accuracy of PMSA and MRI, a study by Emmett et al. in 2021 examined 291 patients, 56% of whom had clinically significant prostate cancer [9]. All patients in this study were triaged via MRI and PI-RADS. Two patients planned to have a biopsy done. The patients underwent PSMA and MRI. It was found that combined PSMA + MRI improved the negative predictive value (NPV) compared with MRI alone (91% vs. 72%, p < 0.001). Sensitivity also improved (97% vs. 83%, p < 0.001); however, specificity was reduced (40% vs. 53%, p = 0.011) [9]. This study shows that PSMA-PET used with MRI can improve the sensitivity of imaging and reduce the number of false negatives; however, it does appear to lower specificity when compared to MRI alone based on these results.

Considering the value of PSMA scans’ potential for development, a study done in 2024 by Luo et al. used 18F-PSMA-1007 PET imaging combined with machine learning technology to predict the chance of SVI after a radical prostatectomy [10]. A retrospective analysis was done examining a sample of 140 prostate cancer patients who had a PSMA PET/CT before a radical prostatectomy. There was a training set of 112 randomly assigned patients from the sample for three different machine learning software programs. The area under the receiver operating characteristic curve (AUC) was calculated for each model regarding its prediction of SVI and a sketch of the volume of interest, with a value of 1 representing perfect diagnostic accuracy. The results of the models were compared with radiologists' assessment, and it was determined that, in the test set, the AUC was higher with all three machine learning models: 0.95, 0.96, and 0.94 vs. 0.70; P = 0.008, 0.004, and 0.002, respectively [10]. PSMA-PET imaging is a relatively new technology, and new methods are being discovered to improve its imaging accuracy. This study shows one way in which this imaging technique can be used to improve accuracy, specifically regarding the chance of SVI.

## Conclusions

Overall, the studies examined show that PSMA-PET imaging with a CT or MRI can improve diagnostic accuracy. Considering the risk of SVI, increasing the diagnostic accuracy of the imaging method could reduce the necessity of treating the area, depending on the results of the scan. In this case, our patient did have high-risk factors, which, based on recommended guidelines, indicated that the seminal vesicles should be treated, and PSMA-PET confirmed that the seminal vesicles were positive. This result stays consistent with current literature on the topic and serves as a demonstration of the accuracy and potential use of PSMA-PET imaging in determining if seminal vesicle treatment is necessary when treating prostate cancer via EBRT.

## References

[1] (2024). American Cancer Society. Key statistics for prostate cancer. https://www.cancer.org/cancer/types/prostate-cancer/about/key-statistics.html.

[2] Yampolsky H (2025). Prostate Cancer Research Institute. Side effects from radiation therapy. https://pcri.org/azure-1/2017/9/18/side-effects-from-radiation-therapy.

[3] Hoffman A, Amiel GE (2023). The impact of PSMA PET/CT on modern prostate cancer management and decision making—the urological perspective. Cancers (Basel).

[4] Salembier C, Villeirs G, De Bari B (2018). ESTRO ACROP consensus guideline on CT- and MRI-based target volume delineation for primary radiation therapy of localized prostate cancer. Radiother Oncol.

[5] Kestin L, Goldstein N, Vicini F, Yan D, Korman H, Martinez A (2002). Treatment of prostate cancer with radiotherapy: should the entire seminal vesicles be included in the clinical target volume?. Int J Radiat Oncol Biol Phys.

[6] Bayman NA, Wylie JP (2007). When should the seminal vesicles be included in the target volume in prostate radiotherapy?. Clin Oncol (R Coll Radiol).

[7] Steed T, Chopra N, Yun J (2023). Seminal vesicle treatment for localized prostate cancer treated with external beam radiotherapy. Curr Oncol.

[8] Chow KM, So WZ, Lee HJ (2023). Head-to-head comparison of the diagnostic accuracy of prostate-specific membrane antigen positron emission tomography and conventional imaging modalities for initial staging of intermediate- to high-risk prostate cancer: a systematic review and meta-analysis. Eur Urol.

[9] Emmett L, Buteau J, Papa N (2021). The additive diagnostic value of prostate-specific membrane antigen positron emission tomography computed tomography to multiparametric magnetic resonance imaging triage in the diagnosis of prostate cancer (PRIMARY): a prospective multicentre study. Eur Urol.

[10] Luo L, Wang X, Xie H (2024). Role of [18F]-PSMA-1007 PET radiomics for seminal vesicle invasion prediction in primary prostate cancer. Comput Biol Med.

